# Nanometric-Scaled Emulsions (Nanoemulsions) 

**Published:** 2010

**Authors:** Reza Aboofazeli

Nanotechnology, shortened to *nanotech*, is the study of the controlling of matter on an atomic and molecular scale. Generally, nanotechnology deals with structures sized between 1 to 100 nanometer in at least one dimension and involves developing materials or devices within that size. The use of nanotechnology in the field of pharmaceutics and drug delivery has grown over the last few years, so remarkably that the pharmaceuticals developed on the basis of this technology are termed as *Nanopharmaceuticals*. Among the nanopharmaceuticals currently being used or under investigation, one can mention nanoemulsions, nanosuspensions, nanospheres, carbon nanotubes, micellar nanocarriers (polymeric micelles), nanocapsules, lipid nanoparticles, self-nanoemulsifying systems, dendrimers, *etc*. 

Nanoemulsions (also referred as miniemulsions, submicron emulsions, ultrafine emulsions, fine-dispersed emulsions and so forth) are a group of dispersed particles used as vehicles for pharmaceutical aims and seem to be promising for the future of cosmetics, diagnosis, drug therapies and biotechnologies. Due to the similarities between microemulsions and nanoemulsions, various definitions have been proposed in the literature, regarding nanoemulsions, as follows: 

Nanoemulsions are non-equilibrium emulsions with a remarkable small droplet size in the range of 20-200 nm, regardless of the preparation method. 

Nanoemulsions are transparent or translucent systems containing droplets with a mean diameter in the range between 100 – 500 nm, and unlike thermodynamically stable microemulsions, they are kinetically stable. 

Nanoemulsions are isotropic and thermodynamically stable dispersions consisting of oil, surfactants, co-surfactants and aqueous phase, usually with a droplet diameter within the range of 10-100 nm. Nanoemulsions are non-equilibrium systems with a spontaneous tendency to separate into the constituent phase, although they may possess a relatively high kinetic stability for a long time, unlike microemulsions which are equilibrium systems (*i.e.* thermodynamically stable). Nanoemulsions can not be formed spontaneously and consequently energy input is required. 

However, some authors prefer to use the word “nanoemulsions” only for those emulsions with droplet size in the nanometer range obtained by high shear methods (*e.g.* high shear stirring, high pressure homogenization and ultrasound generators). These authors believe that the emulsions prepared by methods such as self-emulsifying and phase inversion temperature/composition, although possessing an extremely small droplet size, should not be considered as nanoemulsions, due to the influence of the method of preparation on the droplet size, stability and other emulsion properties. 

As generally accepted, nanoemulsions are transparent or translucent dispersions, having the droplet size of less than 100 nm (the same droplet length-scale as microemulsions) with ultra low interfacial tension, large o/w interfacial areas and long-term physical stability (they are sometimes referred to as “Approaching Thermodynamic Stability”). The following main advantages have made these systems unique and, therefore, attracted much attention for their application in pharmaceutics and drug delivery: 

Higher solubilization capacity, compared to simple micellar solutions, can improve the solubility and bioavailability of hydrophobic compounds. Enormous increase in the interfacial area can influence the transport properties of the drug. 

Brownian motion can keep the droplets from creaming or sedimenting and eventually coalescing. Small droplet size prevents any flocculation, enabling the system to remain dispersed with no separation. Less surfactant concentration is required to prepare nanoemulsion, compared to microemulsions. 

Nanoemulsions are non-toxic and non-irritant and do not damage human and animal cells and hence are suitable for therapeutic purposes. 

Despite having many advantages, the main limitation for developing application of nanoemulsions is their stability. Although it is generally accepted that these systems could remain stable even by years, however, due to the small droplet size, it has been reported that the Oswald ripening could damage nanoemulsions, causing their application to be limited. Therefore, in most cases, nanoemulsions are required to be prepared shortly before their use. In this regard, self-nanoemulsifying drug delivery systems composed of isotropic mixtures of oil, surfactant and co-surfactant with the droplet size in the range of 20 -200 nm, have been recently investigated. These mixtures form fine o/w nanoemulsions upon mild agitation, followed by injection into an aqueous media. Hence, the stability problem is solved by using nanoemulsions immediately after their preparation. 

Literature review reveals that nanoemulsions containing solubilized drugs have been studied extensively as nanocarriers for the treatment of various diseases. Anticonvulsants, antibiotics and antihypertensives are among the drugs solubilized in nanoemulsions. In addition, these nano-scaled structures have been investigated for HIV/AIDS and cancer therapy. The application of nanoemulsions in cosmetics, as a mucosal vaccine, in cell culture technology, in targeted drug delivery, for the improvement of oral delivery of poorly soluble drugs and transdermal delivery are also reported in the literature. 

In conclusion, nanoemulsions could be considered as promising novel formulations for drugs and new chemical entities. In this regard, major advances and developments have been obtained. The presence of nanosized particles (with a large interfacial area), enhanced delivery characteristics, improved biodistribution and pharmacokinetics make these systems appropriate carriers for site-specific drug targeting, especially chemotherapeutic agents.

*Dr. Reza Aboofazeli is currently working as a professor of Pharmaceutics at the Department of Pharmaceutics, School of Pharmacy, Shaheed Beheshti University of Medical Sciences, Tehran, Iran. He could be reached at the following e-mail address: raboofazeli@sbmu.ac.ir.*

